# Differential protection scheme for transmission lines with end-of-line UPFC compensation using sequential current components

**DOI:** 10.1038/s41598-026-48832-5

**Published:** 2026-05-20

**Authors:** Jai Prakash Sharma, Ravi Shankar Tiwari, Om Hari Gupta, Edemialem Gedefaye Mehretie

**Affiliations:** 1 Electrical Engineering Department, GLA University Mathura, Bharthia, UP 281406 India; 2 Electrical Engineering Department, NIT Jamshedpur, Jharkhand, 831014 India; 3 Faculty of Electrical and Computer Engineering, Bahir Dar Institute of Technology, Bahir Dar University, Bahir Dar, Ethiopia

**Keywords:** Differential protection, Dynamic compensation, Sequence current components, Transmission line, UPFC, Energy science and technology, Engineering, Mathematics and computing

## Abstract

With the rapid growth in population, the increase in power demand has outpaced the expansion of transmission line infrastructure, often leading to overload conditions. To mitigate such challenges, Flexible AC Transmission System (FACTS) devices are widely adopted to enhance system reliability and stability. While FACTS devices significantly boost the power transfer capability of transmission lines, they can also impact the effectiveness of traditional protection relays. Among these, the Unified Power Flow Controller (UPFC) is particularly notable for its ability to regulate both active and reactive power flows independently, offering the advantages of combined series and shunt compensation. This study introduces a protection strategy based on differential current sequence components for a transmission line equipped with end-line UPFC compensation (ELUC). The proposed algorithm is validated through simulations in MATLAB Simulink and further assessed using a real-time simulator. The evaluation considers a range of scenarios including variation in fault types, fault locations, fault resistances, UPFC operating modes, simultaneous faults, and synchronization errors. The performance is thoroughly examined through real-time simulation and benchmarked against existing techniques. The results demonstrate that the proposed scheme is both selective and accurate in detecting faults under dynamic conditions.

## Introduction

In order to meet the continuous growing power demand over a system, existing transmission line networks (TLNs) are operated at their maximum limits^1–5^. To increase the reliability and stability of the system, FACTS devices are used for compensation^6–11^. Compared to static compensation, dynamic compensation provides better control to maintain voltage regulation and change in system frequency. To have a concurrent control over both real and reactive power flow, UPFC is used in TLN. UPFC is a combination of series compensating device i.e. SSSC and shunt compensating device i.e. STATCOM. Such dynamic hybrid compensation creates challenges with conventional protective relaying^12^. Hence there is a requirement to have an algorithm that can detect fault in presence of UPFC in TLN.

Although in literature there are a lot of schemes available for fault detection in TLN with dynamic compensation; however, these schemes have some limitations in their performance. In^13^, adaptive distance protection is based on the phase component of three voltages and currents at the sending end and fault points. The authors formulate an approach to eliminate the effect of UPFC and compensate for fault resistance. However, the method is tested for limited fault resistance (40 Ω) and fault scenario (ground faults only), reliance on accurate measurement and may encounter sub-reaching during high impedance faults. In^14^, the normalized value of superimposed currents is used to detect and classify the faults in a UPFC-compensated line. Conversely, the fault classification depends on the decision-try approach, thus, difficult to set appropriate thresholds for each level of the decision tree causing misclassification. Additionally, the UPFC dynamics may change the line impedance causing false detection because it is not always feasible to adopt these changes in real-time applications. Susceptibility to power swing and load encroachment are the other challenges in distance relaying used for lines consisting of FACTS and renewable integration^15^. Fault characteristics are not identified by traditional reclosing schemes in a UPFC-compensated line, causing a second restrict due to reclosing for permanent faults. Reference^16^ adopts an active injection of characteristic voltage by UPFC control to identify faults. This distinguishes the voltage and current characteristics between temporary and permanent to prevent reclosing during permanent faults. The presence of UPFC also causes dynamics in fault current, leading to the mal-operation of unit protection schemes. To overcome this, a short-term matrix pencil method (STMPM) constitutes the per-phase differential current and analyze the net change in current magnitude using first and second-order derivatives to identify faults^17^. But, uncertainty in real-time execution and computational complexity in STMPM are the major challenges.

Discrete wavelet transform together with probabilistic neural network are used to detect, identify and locate faults in^18^. A hardware in loop simulation to detect, classify and locate faults in UPFC compensated lines is given in^19^. In^20^, the K-nearest neighbouring (KNN) machine learning technique is used to detect and classify faults in UPFC-compensated lines. The fundamental current and voltage components are extracted from the time domain signal for different scenarios using discrete Fourier transform are used to train KNN. However, the selection of window size, processing time, need of diversified training data and computational complexity are the challenges in the real-time implementation of this scheme.

To abate the aforementioned limitations, an algorithm based on differential sequence current components (DSCC) has been discussed in this article. The scheme is scrutinized and validated under various concerned scenarios by using the MATLAB Simulink environment. Followings are the key highlights/contributions of the proposed DSCC-based scheme:-

- The DSCC-based scheme remains selective and accurate, for the fault detection with variation in the fault distance; change in the fault resistance, simultaneous faults and measurement noise.
- The proposed scheme shows promising performance with the sample synchronization error of 7.2° i.e. 327 times more than the tolerable limit.
- The DSCC-based protection criterion is inherently robust to UPFC operating modes.

Furthermore, the performance of the proposed algorithm is also analyzed by using an Opal-RT 4510 real-time simulator, followed by a detailed comparative analysis with the latest available schemes in the literature.

## Proposed

**Fig. 1:**
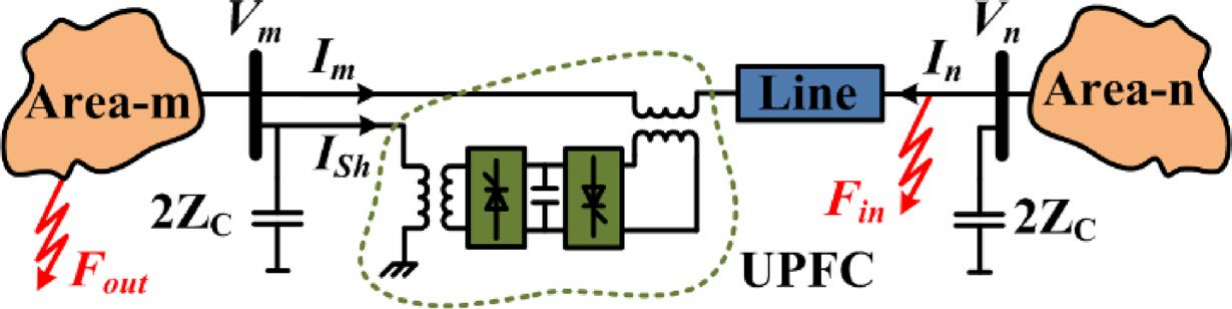
Equivalent block diagram of a two-area transmission line system with ELUC.

In order to understand differential sequence current component (DSCC)-based scheme, let us consider a transmission line system with ELUC as depicted in Fig. 1. TLN with the π-model is considered. Faults F_in_ and F_out_ are considered as internal and external faults respectively. The 3-phase differential current of the TLN as shown in Fig. 1, is calculated using (1), as given below:

1 $$I_{diffx} = I_{mx} + I_{nx} - \frac{{\left( {V_{mx} + V_{nx} } \right)}}{{2Z_{Cx} }} - I_{shx}$$

where, *I*_*m*_ = Sending end current, *I*_*n*_ = Receiving end current, *I*_*sh*_ = Shunt component of current being drawn by UPFC,* I*_*diff*_ = differential current, *Z*_*C*_ = Impedance of coupling capacitance, and *x* = *a, b, c* for phase-a, phase-b and phase-c respectively.

Now, the symmetrical sequence component of differential current (SSDC) can be calculated using (2),(3),(4), as given below:

2 $$I_{0} = {{\left( {I_{diffa} + I_{diffb} + I_{diffc} } \right)} \mathord{\left/ {\vphantom {{\left( {I_{diffa} + I_{diffb} + I_{diffc} } \right)} 3}} \right. \kern-0pt} 3}$$

3 $$I_{1} = {{\left( {I_{diffa} + \lambda I_{diffb} + \lambda^{2} I_{diffc} } \right)} \mathord{\left/ {\vphantom {{\left( {I_{diffa} + \lambda I_{diffb} + \lambda^{2} I_{diffc} } \right)} 3}} \right. \kern-0pt} 3}$$

4 $$I_{2} = {{\left( {I_{diffa} + \lambda^{2} I_{diffb} + \lambda I_{diffc} } \right)} \mathord{\left/ {\vphantom {{\left( {I_{diffa} + \lambda^{2} I_{diffb} + \lambda I_{diffc} } \right)} 3}} \right. \kern-0pt} 3}$$

where *λ* = 1∠120°.

Now the above SSDC as given in (2), (3), and (4) are used to obtain a criterion coefficient (CC) as given in (5), below:

5 $${\mathrm{CC}} = \frac{{|I_{0} + I_{2} |}}{{|I_{1} |}}$$

A. CC for different fault types

In a power system, the fault types mainly classified as single line fault (LG), double line fault (LL), double line to ground fault (LLG), and symmetrical fault (LLL or LLLG faults). Now, CC is obtained for different faults as given below:

- For an LG fault, *I*_*1*_ is less than *I*_*d*(max)_ (where, *I*_*d*(max)_ = Max(*I*_*diffx*_)), and all 3 SSDC are equal to each^21^ and can be expressed as given in (6), below:
  6 $$I_{0} = I_{1} = I_{2}$$

Therefore, the CC for LG fault is as follow in (7):

7 $${\mathrm{CC}} = 2$$

- Similarly for LL fault, *I*_*1*_ is again less than *I*_*d*(max)_, zero SSDC is absent^22^, and positive and negative SSDC are equal in magnitude as given in (8), below:
  8 $$|I_{1} | = |I_{2} |$$

Therefore, the CC for LL fault is as follow in (9):

9 $${\mathrm{CC}} = 1$$

- For LLG fault, *I*_*1*_ is again less than *I*_*d*(max)_, and the relationship between SSDC is given in (10), below:
  10 $$|I_{0} + I_{2} | = |I_{1} |$$

Therefore, the CC for LL fault is as follow in (11):

11 $${\mathrm{CC}} = 1$$

- From the evaluation of the above two cases, it can be perceived that the values of CC are same for the both cases, however, *I*_*0*_ is present in case of LLG whereas absent in case of LL faults.
  Now, for LLL or 3-phase fault, *I*_*1*_ is equal to *I*_*d*(max)_, and there is only positive SSDC is present in TLN^23^. Therefore, the CC for 3-phase fault is as follow in (12):
  12 $${\mathrm{CC}} = 0$$

On analysis, the nature of CCs for different faulty cases, it can be perceived that CC along with zero SSDC can be used for fault classification. Now to have the main function of a relaying algorithm, i.e., to discriminate between an internal fault from the normal operating conditions and the external disturbances; the magnitudes of *I*_*0*_ and *I*_*2*_ are used. Since, *I*_*0*_ and *I*_*2*_ are available during the unbalanced conditions and their differential magnitudes are large in case of internal faults. So, before initiating the classification algorithm based on CC and zero SSDC; a warning signal (War.) (based on the rigorous analysis of real-time simulation results) is generated as per (13) below:

13 $$War.\left\{ {\begin{array}{*{20}c} { = 1;\,\forall \, I_{i} \ge (0.25 \times {\mathrm{I}}_{{{\mathrm{TH}}}} )} \\ { = 0;\,\forall \, I_{i} < (0.25 \times {\mathrm{I}}_{{{\mathrm{TH}}}} )} \\ \end{array} } \right.$$

where *i* = 0 or 2 for zero or negative SSDC respectively and I_TH_ = 2*I*_*dpre*_ (i.e. twice the pre-fault differential current).

B. Relaying scheme

**Fig. 2:**
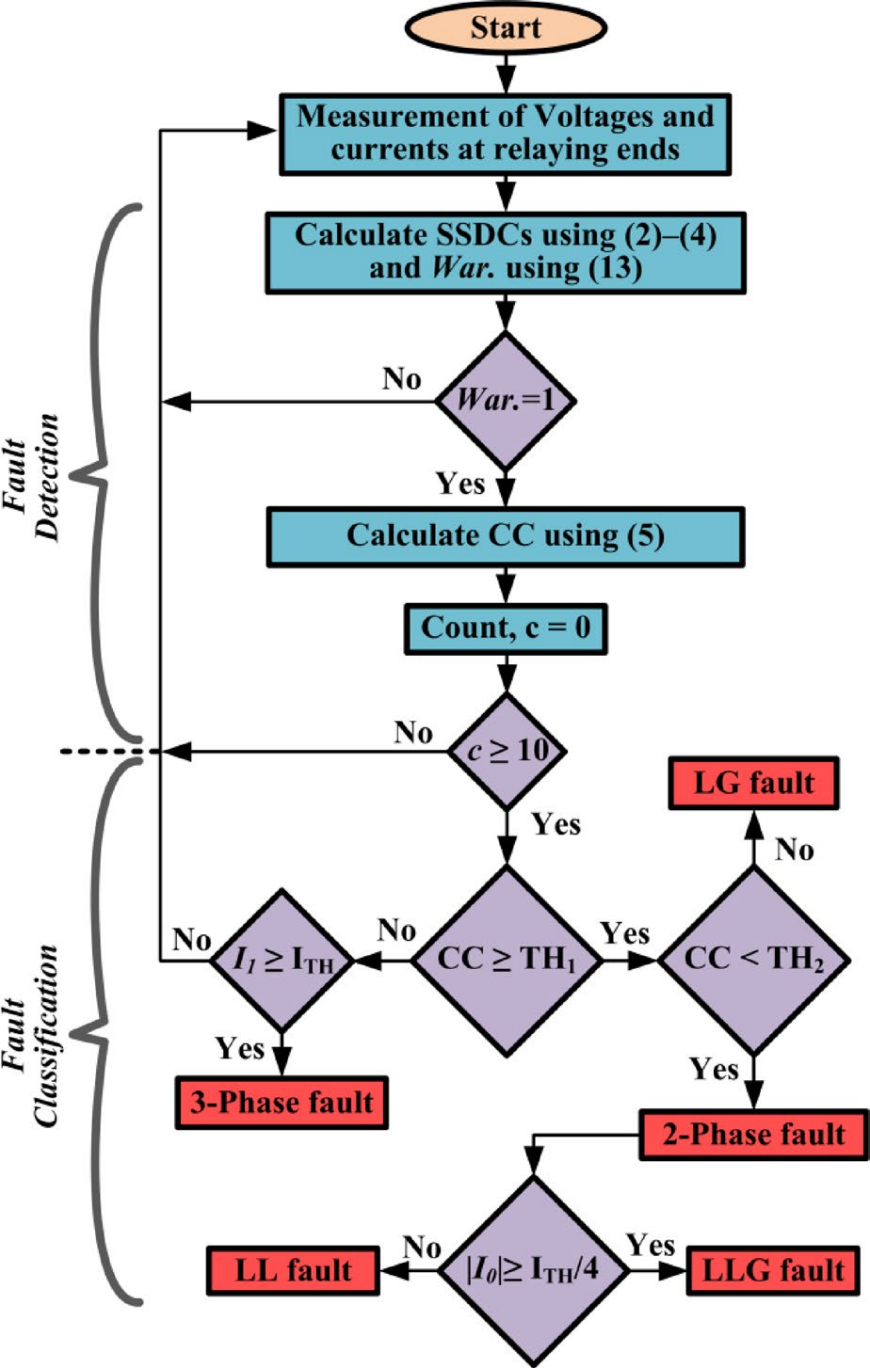
Flow-diagram of DSCC-based algorithm.

**Fig. 3:**
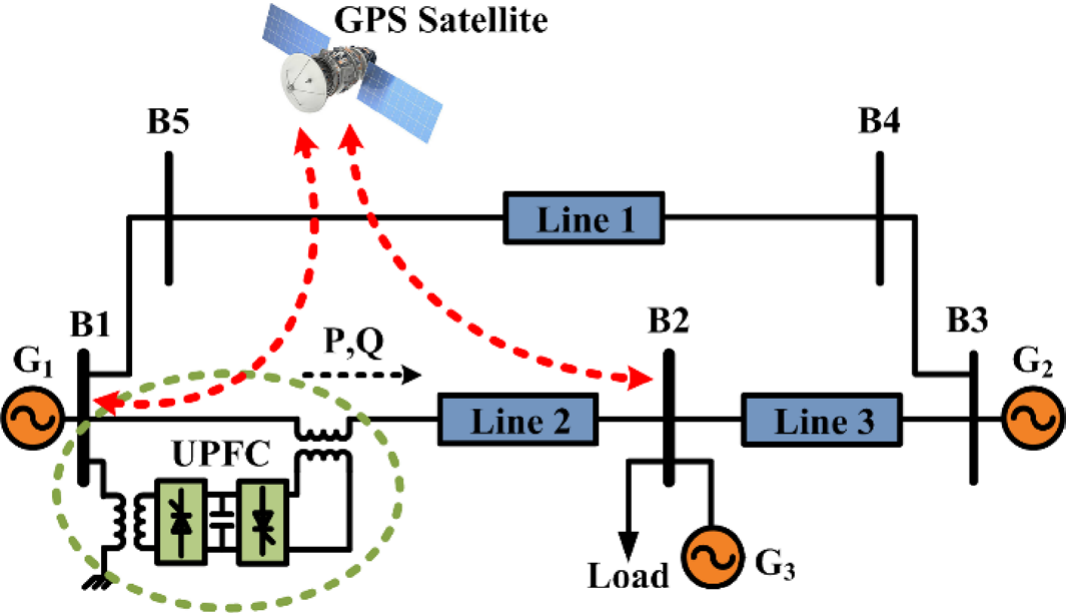
Single line diagram of the considered test system.

The flowchart of the DSCC-based algorithm is manifested in Fig. 2. Firstly, the voltage and the current GPS time-synchronized signals are recorded at the both relaying ends as shown in Fig. 3. After that SSDC and *War.* signal are calculated using Eqs. (2)–(4) and (13). If *War.* is found to be equal to unity; CC is calculated using Eq. (5). Also a counter c, is used to eliminate the influence of transient spikes on the overall algorithms. The counter starts counting when *War.* is high and if the count c is equal or more than 5 then CC is compared with first threshold value (TH_1_). If it is found to be less than TH_1_, then *I*_*1*_ is compared with I_TH_. If *I*_*1*_ is found to be equal and more than I_TH_, a 3-phase fault identified, else it is perceived as external disturbance. However, if CC is found to be equal and more than TH_1_, then it is again compared with second threshold value TH_2_. If CC is found to be less than TH_2_, an LG fault is identified; otherwise it is perceived as 2-phase fault. In order to distinguish between LL and LLG fault *I*_*0*_ is compared with I_TH_/4. If *I*_*0*_ is found more than I_TH_/4, a LLG fault is identified, else LL is perceived.

C. Threshold selection

In the DSCC-based algorithm a total of three quantities is compared with respective threshold values. Based on a rigorous evaluation of different transmission-line configurations and fault scenarios, a 20% safety margin was incorporated to ensure robustness. Consequently, the lower threshold corresponding to unsymmetrical faults is selected slightly below the ideal value (i.e. TH_1_ ≈ 0.9), while the upper threshold (i.e. TH_2_ ≈ 1.5) is chosen as the mean value between the theoretical limits for single-phase and two-phase faults, ensuring maximum separation and clear discrimination at the classification boundary. The threshold for *I*_*1*_ is kept as a twice of the pre-fault differential current value, i.e., I_TH_ = 2*I*_*dpre*_^24^.

## Results and discussions

A 5-bus transmission network with ELUC as shown in Fig. 3 is used as a test system. The GTO-based UPFC is of 48-Pulse, 500 kV and 100 MVA rating and is used to control the power flow in a 75 km Line 2. So, the Line 2 is considered as the protected line for algorithm performance analysis. The length of Line 1 is 200 km and Line 3 is 180 km. The complete test system details are provided in Appendix 1. The UPFC is connected at bus B1. One of the UPFC converters is connected in shunt at bus B1 and another is connected in series between buses B1 and B2. The shunt and the series converters can exchange power through a common DC bus.

### Performance analysis with an internal LG fault

As AG fault is the most probable fault in an electrical power system, so the performance of the proposed algorithm has been examined with variation in fault distance (FD), fault resistance (FR), also with simultaneous faults, presence of noise in measurement and internal fault with sample synchronization error (SSE).

#### Performance analysis with variation in FD

Protection algorithm may get affected due to variation in FD, so the performance of the proposed scheme has been examined with variation in FD from Bus-1, for an internal AG fault in Line 2 with FR = 10 Ω. The obtained results are listed in Table 1 below

**Table 1:** Variation in FD for an LG fault.

FD (in km)	WT (in ms)	DT (in ms)	CC	I_1_ (in kA)	I_0_ (in kA)
0	2.91	3.167	2.15	0.243	0.108
25	2.91	3.08	2.12	0.246	0.11
35	2.83	2.91	2.11	0.248	0.109
40	2.83	2.91	2.10	0.252	0.11
50	2.83	2.91	2.09	0.260	0.124
75	2.66	2.75	2.05	0.278	0.147

**WT = Warning signal generation time

**DT = Detection time

**Fig. 4:**
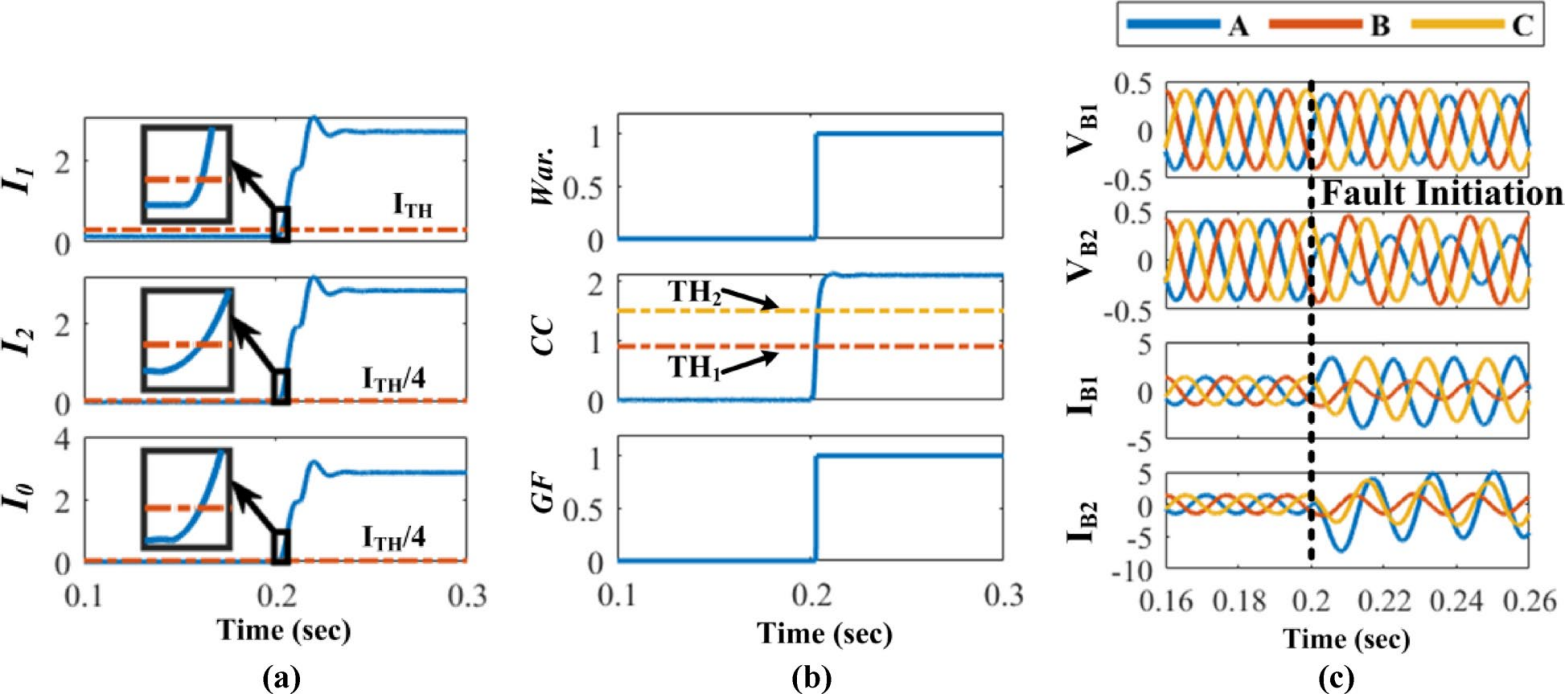
For an AG fault in line-2 with FR = 10Ω at FD = 35 km from Bus-1 (**a**) SSDC, (**b**) Relaying criterion coefficients, and (**c**) 3-phase voltages and currents at Bus-1 and Bus-2.

For an internal fault in Line-2 with FD = 35 km from Bus-1, the Fig. 4 manifests different criterion coefficients, differential currents and 3-phase voltages-currents waveforms of Bus-1 and Bus-2. *I*_*0*_ and *I*_*2*_ have crossed the threshold (I_TH_/4) as shown in from Fig. 4a; as a result, the warning signal is high (i.e. *War.* = 1). Also, from Fig. 4b, it can be inferred that CC > TH_1_ and CC > TH_2_; so, the fault is diagnosed as LG fault. Since the fault involves ground, the Ground Fault (GF) signal is latched to high (i.e. *GF* = 1), as illustrated in Fig. 4b.

#### Performance analysis with change in FR

**Fig. 5:**
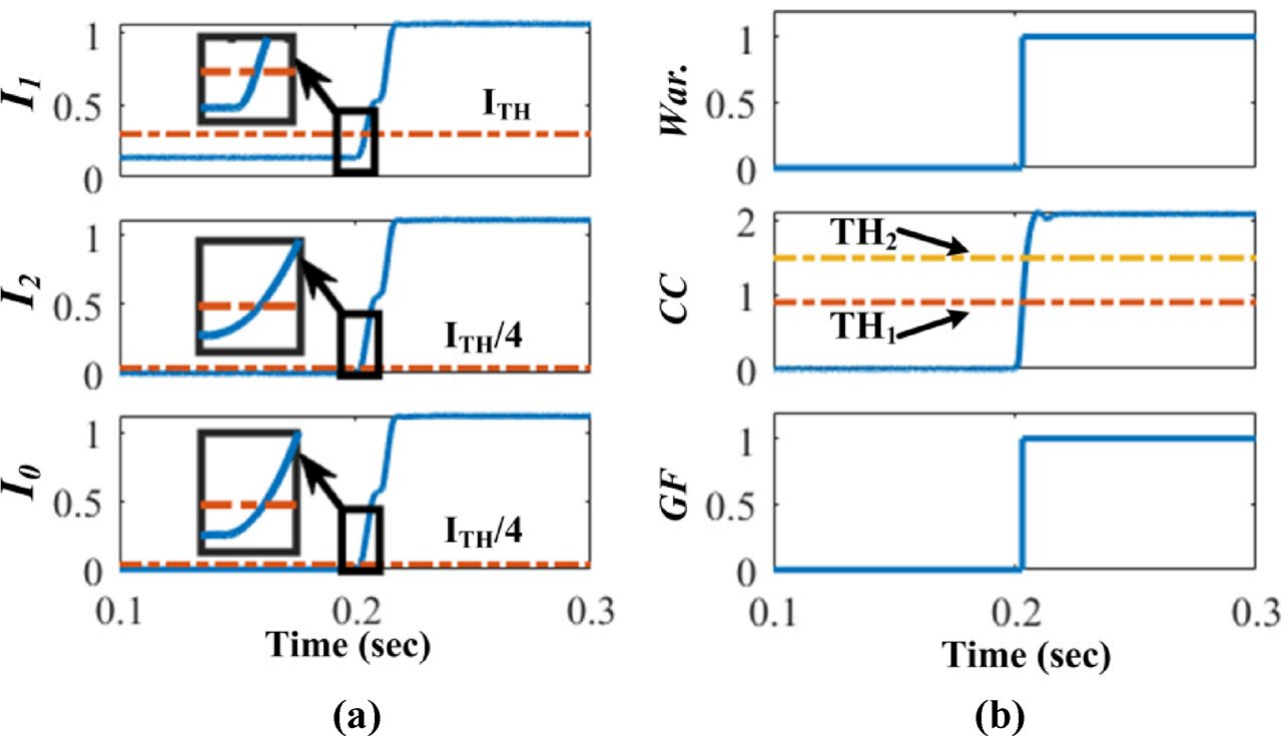
For an AG fault in line-2 with FR = 100Ω at FD = 30 km from Bus-1 (**a**) SSDC, and (**b**) Relaying criterion coefficients.

In order to witness the impact of FR variation in the proposed algorithm, a single type of fault is introduced (i.e. an LG fault) at 30 km from Bus-1 with different values of FR and the obtained results are listed in Table 2. For an AG fault in line-2 with FR = 100Ω at FD = 30 km from Bus-1, Fig. 5, presents the SSDC and relaying criterion coefficients. The warning signal is *War.* = 1(high); as* I*_*0*_ and *I*_*2*_ have crossed the threshold (I_TH_/4) as manifested in Fig. 5a. The fault is identified as the LG fault as CC > TH_1_ and CC > TH_2_ as shown in Fig. 5b. Due to increase in the FR the magnitude of the SSDC is very small compared to lower value of FR, however, the algorithm remain unaffected, selective and accurate for fault detection.

**Table 2:** Variation in FR for an LG fault.

FR (in Ω)	WT (in ms)	DT (in ms)	CC	I_1_ (in kA)	I_0_ (in kA)
0	2.83	2.91	2.09	0.241	0.123
15	2.83	2.91	2.09	0.23	0.112
30	2.91	3.00	2.09	0.228	0.112
60	3.00	3.08	2.08	0.219	0.103
75	3.00	3.08	2.08	0.211	0.096
100	3.08	3.16	2.08	0.207	0.093

#### Performance under simultaneous faults

To have the simultaneous faults, an LG fault is created at 25 km from Bus-1 (i.e. an internal fault in Line 2) and an LLG fault is introduced at 30 km from Bus-2 (i.e. an external fault in Line 3).

**Fig. 6:**
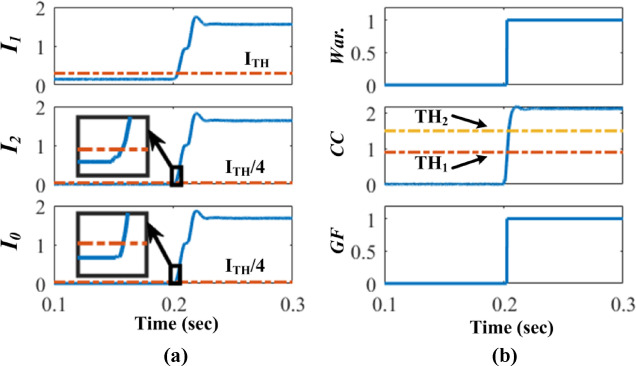
For a simultaneous faults (**a**) SSDC, and (**b**) Relaying criterion coefficients.

The performance parameters of the proposed algorithm are manifested in the Fig. 6. After, *War.* becomes high (i.e. *I*_*0*_ and *I*_*2*_ have crossed the threshold (I_TH_/4)); it can be seen from Fig. 6b; that CC > TH_1_ and CC > TH_2_. So, it can be concluded that the algorithm remains selective and accurate for simultaneous fault as well. Hence, the inspected fault is identified as LG fault.

#### Performance analysis with measurement noise

In the proposed scheme a GPS-time tagged signals are being measured at both ends of the protected transmission line. Hence, it is very important to study the impact of measurement noise in the proposed algorithm. The real time noise in the measurement by PMUs are the Gaussian type with a signal-to-noise ratio (SNR) range of 30–60 dB as per the references^25,26^. In order to see the impact of measurement noise on the scheme an AG fault is created on Line-2 with a fault resistance (FR) of 100 Ω at a fault distance (FD) of 45 km from Bus-1 at 0.2 s. After that white Gaussian noises are intentionally added to the measured signals from the both ends of the transmission line. The performance of the scheme is studied with a variation of SNR in the range of 30–60 dB and the same are listed in the Table 3, below.

**Table 3:** Variation in FD for an LG fault.

SNR (in dB)	WT (in ms)	DT (in ms)	CC	I_1_ (in kA)	I_0_ (in kA)
30	3.08	3.25	2.09	0.238	0.104
40	3.08	3.33	2.08	0.242	0.112
50	3.08	3.33	2.08	0.241	0.110
60	3.08	3.33	2.08	0.241	0.111

**Fig. 7:**
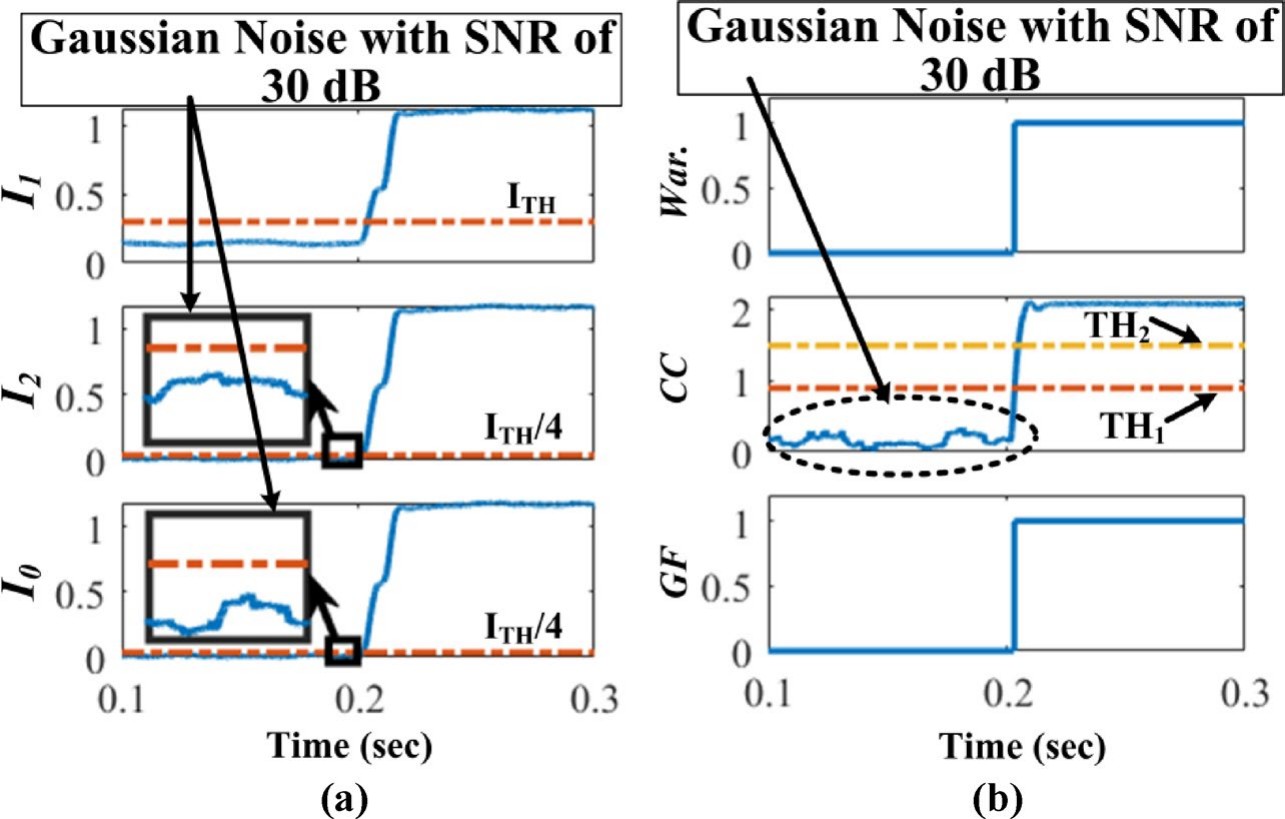
For an LG fault with noise in measurement of 30 dB (**a**) SSDC, and (**b**) Relaying criterion coefficients.

The performance parameters of the proposed scheme with a noise of SNR 30 dB are shown in the Fig. 7. The presence of measurement noise is clearly visible in Fig. 7a and b. However, *War.* becomes high as *I*_*0*_ and *I*_*2*_ have crossed their respective threshold (I_TH_/4) values as manifested Fig. 7a and b. Finally, from Fig. 7b it can be seen that the fault is identified as an LG fault as the CC > TH_1_ and CC > TH_2_. So, it can be concluded that the algorithm remains selective and accurate with the presence of noise in the measurement as well.

#### Performance with sample synchronization error (SSE)

**Fig. 8:**
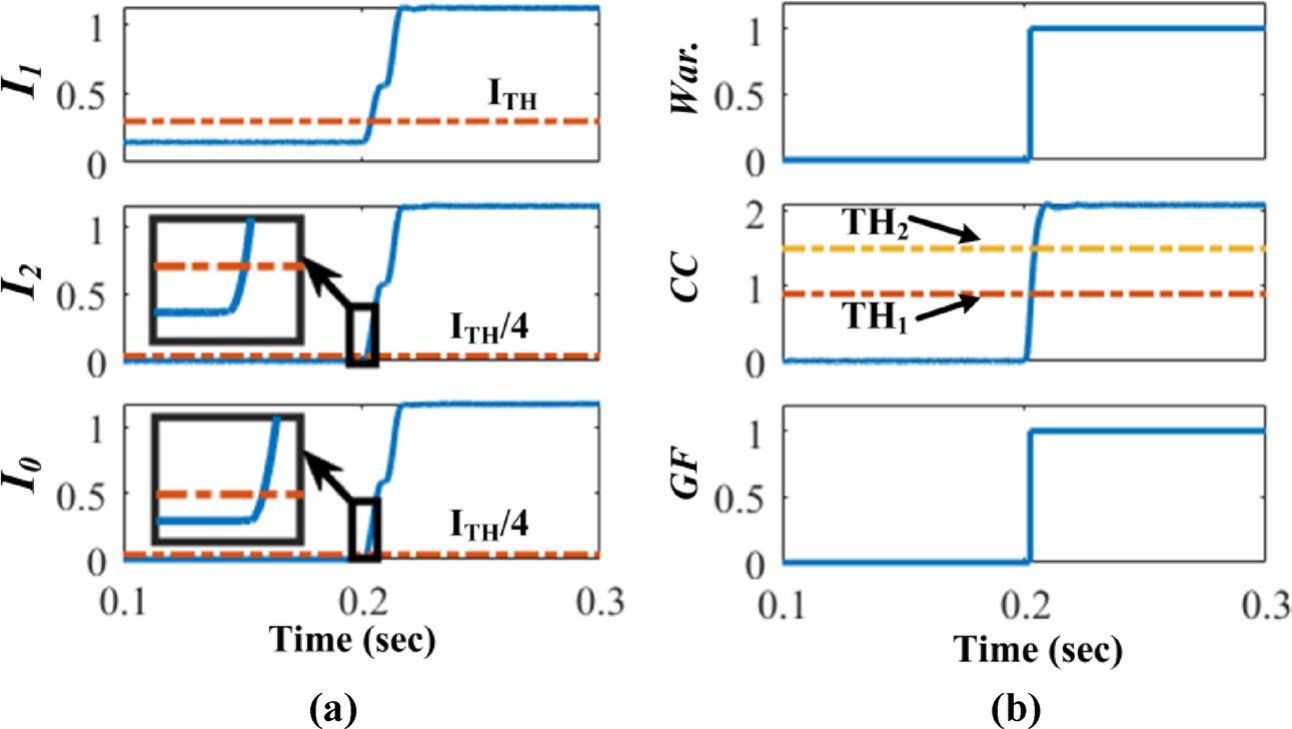
For an LG fault with SSE (**a**) SSDC, and (**b**) Relaying criterion coefficients.

As in the proposed method, GPS time-tagged data from both ends being used for fault identification; hence it is very important to scrutinize the performance of the algorithm with SSE underneath the permissible limit (i.e. 0.022° for a 60 Hz system as per^27^). An LG fault is introduced at 50 km from Bus-1 with a SSE of 7.2° i.e. 327 times more than the tolerable limit. The obtained result is shown below in the Fig. 8. It can be seen that the proposed algorithm remain unaffected with SSE.

### Performance analysis under the internal LL and LLG faults

The second most probable fault in a power system is the LL fault and the third most is the LLG fault. With variation in FD and FR, different types of LL and LLG internal faults are introduced in the Line-2 and the obtained result is shown in the Table 4.

**Table 4:** Obtained results with different types of LL and LLG faults.

Variation in FD with FR of 10 Ω						
FT	FD (in km)	WT (in ms)	DT (in ms)	CC	I_1_ (in kA)	I_0_ (in kA)
AB	0	1.33	1.41	1.01	0.376	0
BC	25	1.16	1.25	1.01	0.326	0
CA	35	1.33	1.41	1.01	0.056	0
ABG	40	1.41	1.5	1.02	0.351	0.077
BCG	50	1.16	1.25	1.02	0.329	0.039
CAG	75	1.25	1.33	1.01	0.144	0.043

Variation in FR at FD of 50 km from Bus-1						
FT	FR (in Ω)	WT (in ms)	DT (in ms)	CC	I_1_ (in kA)	I_0_ (in kA)
AB	0.01	2.91	3.167	2.15	0.243	0.108
BC	1	2.91	3.08	2.12	0.246	0.11
CA	3	2.83	2.91	2.11	0.248	0.109
ABG	5	2.83	2.91	2.10	0.252	0.11
BCG	10	2.83	2.91	2.09	0.260	0.124
CAG	30	2.66	2.75	2.05	0.278	0.147

**Fig. 9:**
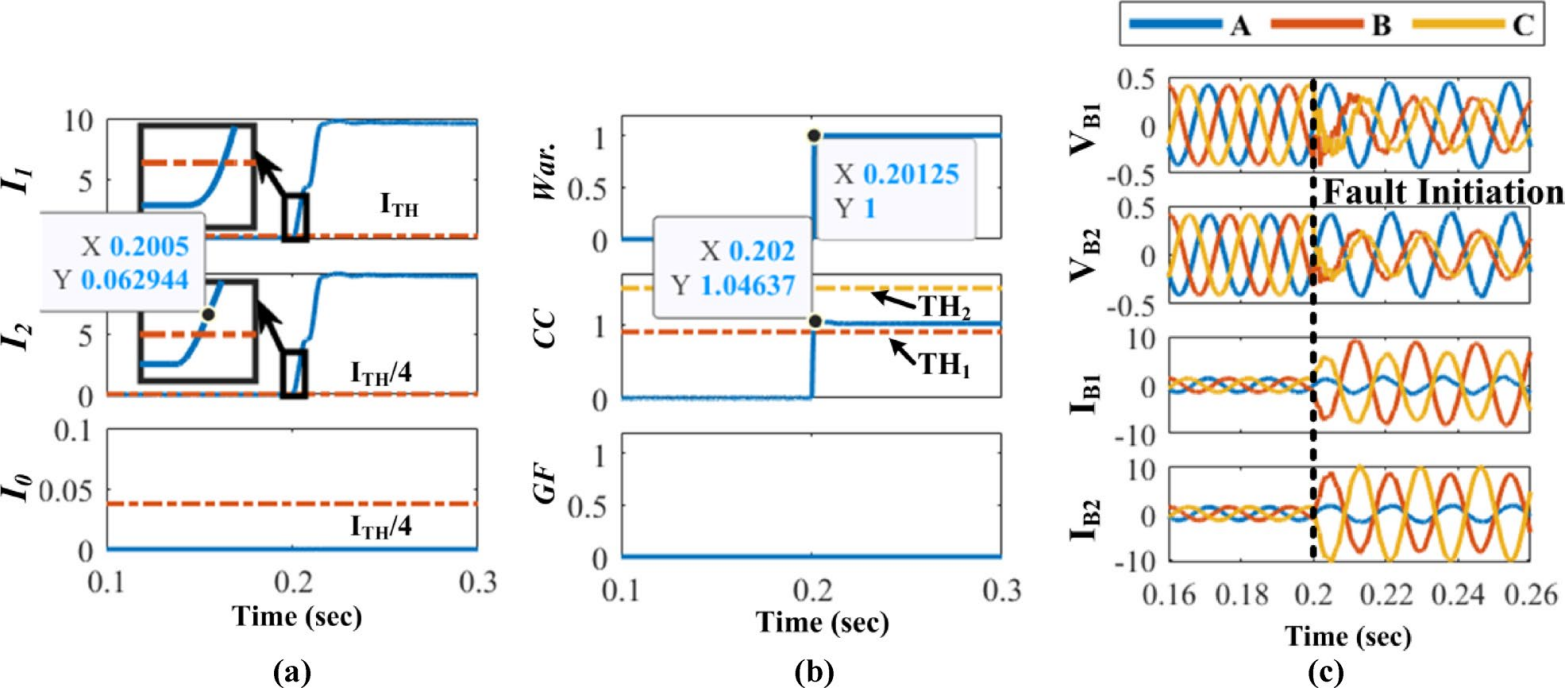
For a BC fault in line-2 with FR = 1Ω at FD = 50 km from Bus-1 (**a**) SSDC, (**b**) Relaying criterion coefficients, and (**c**) 3-phase voltages and currents at Bus-1 and Bus-2.

A BC fault is introduced in line-2 with *R*_*f*_ = 1Ω at FD = 50 km from Bus-1 and the response of the algorithm is shown in the Fig. 9 below. From Fig. 9a, it can be inferred that *I*_*2*_ has crossed the threshold (I_TH_/4); *War.* = 1(high). The fault is identified as 2-phase fault as CC is greater than TH_1_ but less than TH_2_ as shown in Fig. 9b. However, as *I*_*0*_ has not crossed the threshold (I_TH_/4); *GF* = 0(low), indicating there is no ground involved with the fault. Hence, the fault is found to be a LL fault.

**Fig. 10:**
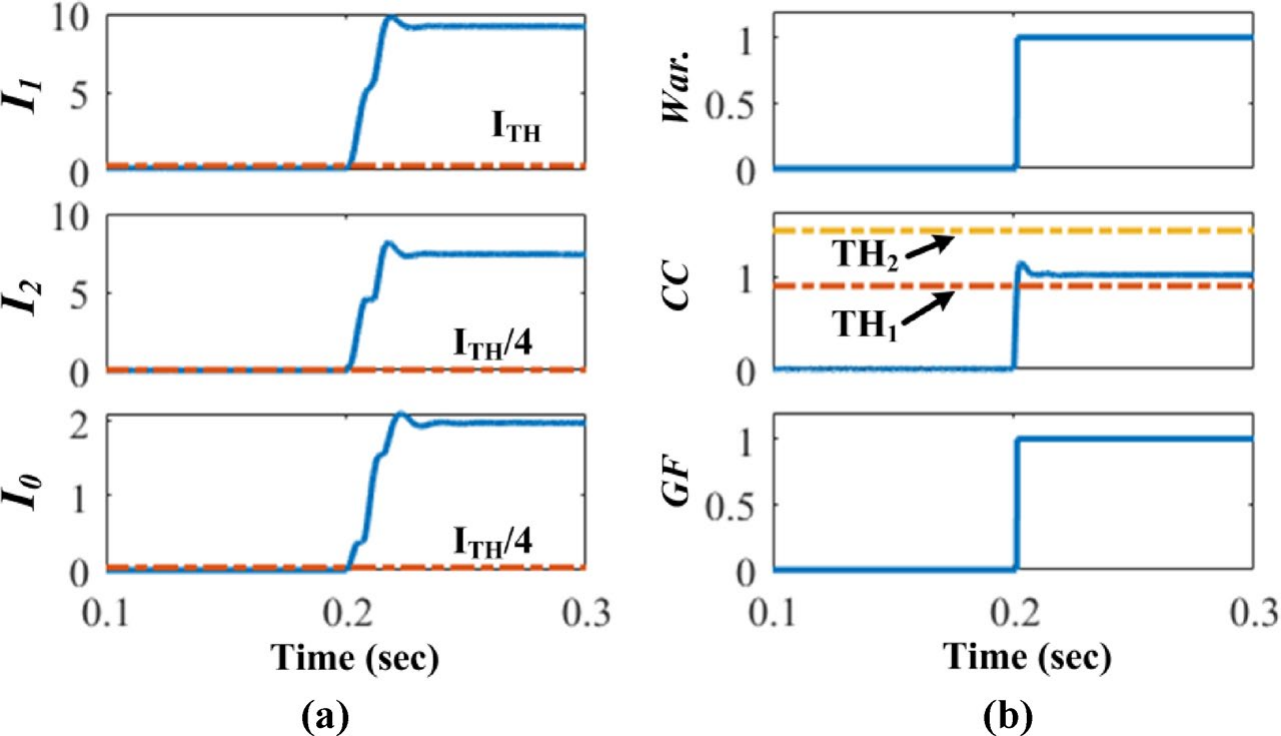
For a ABG fault in line-2 with FR = 5Ω at FD = 50 km from Bus-1 (**a**) SSDC and (**b**) Relaying criterion coefficients.

Similarly, for an ABG fault in line-2 with *R*_*f*_ = 5Ω at FD = 50 km from Bus-1; various relaying aspects and the proposed algorithm’s responses are manifested in Fig. 10. *War.* = 1(high) as *I*_*0*_ and *I*_*2*_ have crossed the threshold (I_TH_/4) as depicted in the Fig. 10a. Again, the fault is recognized as 2-phase fault as CC > TH_1_, but CC < TH_2_ as manifested in Fig. 10b. As a result, the value of *I*_*0*_ has been compared with the threshold value I_TH_/4. It can be seen from Fig. 10a, that *I*_*0*_ has crossed the threshold value; *GF* = 1(high), indicating there is a ground involved with the fault. Hence, the fault is determined as a LLG fault.

### Performance analysis under an internal 3-phase fault

**Fig. 11:**
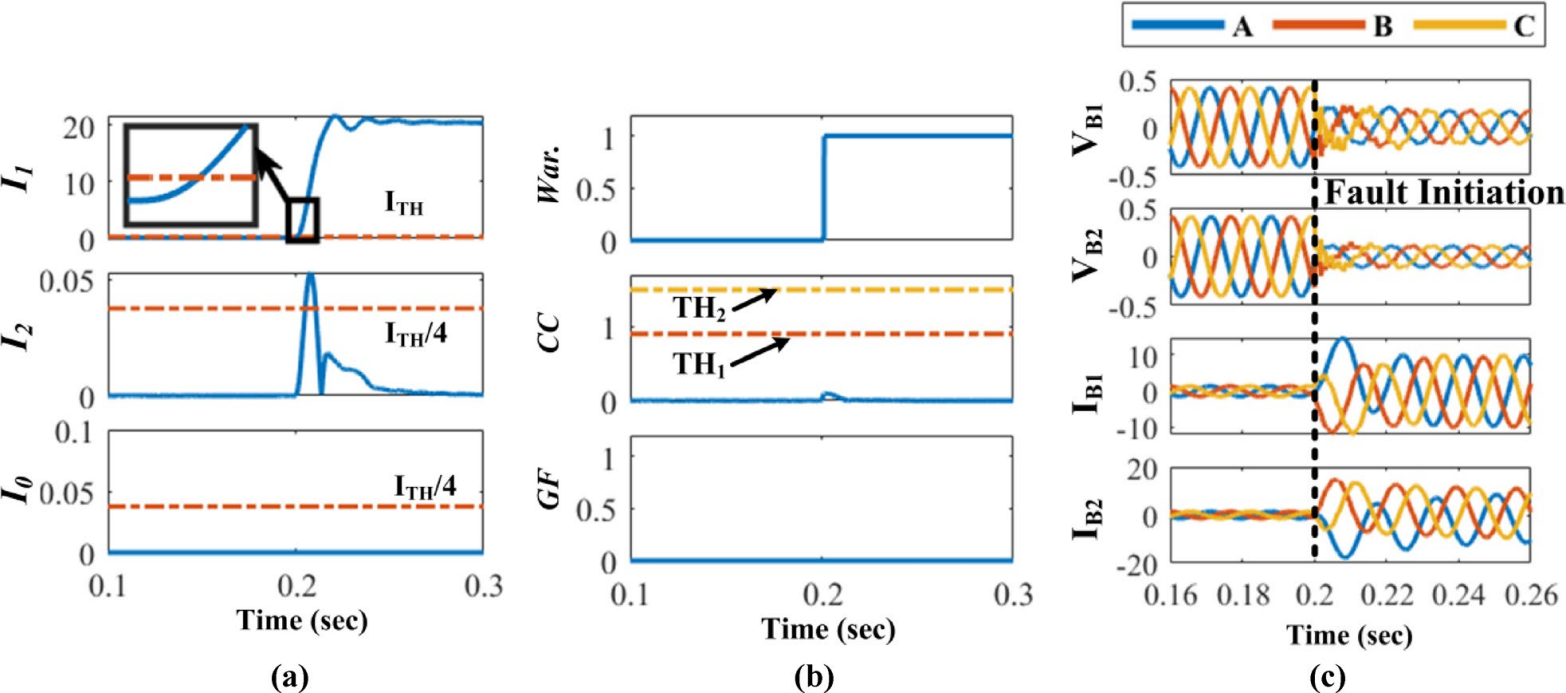
For a 3-phase fault in line-2 with FR = 0.001Ω at FD = 45 km from Bus-1 (**a**) SSDC, (**b**) Relaying criterion coefficients, (**c**) 3-phase voltages and currents at Bus-1 and Bus-2.

The least probable fault in a power system is the 3-phase fault; however, it is the most chaotic fault. Hence, it is very important to examine the performance of the proposed scheme for a 3-phase internal fault. For that, a 3-phase fault is introduced at 45 km away from B1 with *R*_*f*_ = 0.001 Ω and corresponding result is shown in Fig. 11 below. As per theory for a 3-phase fault; the positive SSDC is only present in TLN. However, due to sudden change in the system (i.e. an internal fault) a spike of negative SSDC is found it in TLN. This spike is sufficient enough to trigger the warning signal (i.e. *War.* = 1) as depicted in Fig. 11a. It can be seen from Fig. 11b that, the value of CC is less than TH_1_. Therefore, the magnitude of *I*_*1*_ has been compared with I_TH_, and found to be more than that. So, the fault is identified as a 3-phase fault.

### Performance analysis under variation of operating modes of UPFC

**Fig. 12:**
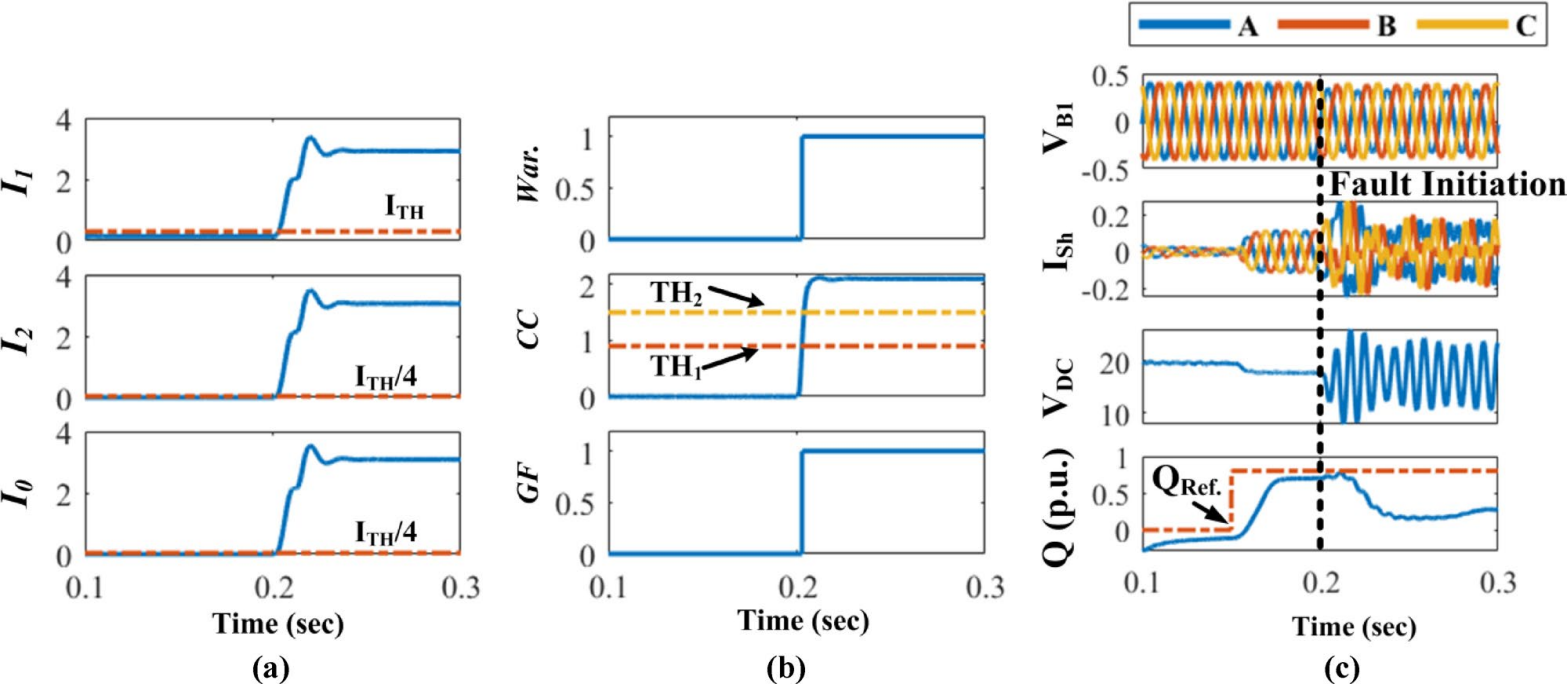
For an AG fault in line-2 with FR = 10Ω at FD = 35 km from Bus-1 (**a**) SSDC, (**b**) Relaying criterion coefficients, (**c**) 3-phase voltages at Bus-1, 3-phase shunt currents and DC link voltage (V_DC_) of UPFC, and reactive power in p.u. of line-2.

As UPFC is one of the dynamic FACTS device, it is essential to scrutinize the performance of the proposed algorithm with different operating modes of the UPFC. In order to analyze the impact of change in operating modes of UPFC, an LG fault is created in Line-2 with *R*_*f*_ = 10 Ω and FD = 35 km from Bus-1 and responses of the scheme are observed; which are listed in Table 5 as well. The operating mode of the UPFC is altered by adjusting the concerned reference values at 0.15 s., with a fault introduced at 0.2 s. For the operating mode STATCOM with Var control; the reactive power is changed from 0 p.u. to + 0.8 p.u. at 0.15 s as depicted in Fig. 12c.

**Table 5:** Variation of operating modes of UPFC for an LG fault.

UPFC operating mode	WT (in ms)	DT (in ms)	CC	I_1_ (in kA)	I_0_ (in kA)
SSSC (Voltage Injection)	2.75	2.83	2.10	0.253	0.113
STATCOM (Var Control)	2.83	2.91	2.10	0.255	0.117
STATCOM (Voltage Control)	2.83	2.91	2.10	0.256	0.117
STATCOM (Var Control) + SSSC (Voltage Injection)	2.75	2.83	2.10	0.253	0.113
UPFC (Power Flow Control)	2.91	3.00	2.10	0.259	0.121

Figure 12c clearly illustrates that the reactive power in Line-2 varies with changes in the reference value, indicating that the system operates under dynamic compensation. The response of the proposed algorithm under dynamic compensation is shown in Fig. 12 below. The warning signal (*War.* = 1) is triggered when *I*_*0*_ and *I*_*2*_ exceed the threshold level (I_TH_/4), as illustrated in Fig. 12a. The fault is classified as an LG fault since CC surpasses both TH_1_ and TH_2_, as shown in Fig. 12b. This confirms that the algorithm remains unaffected by the variation in UPFC operating modes and maintains its selectivity and accuracy in fault detection.

### Performance analysis under external faults

**Fig. 13:**
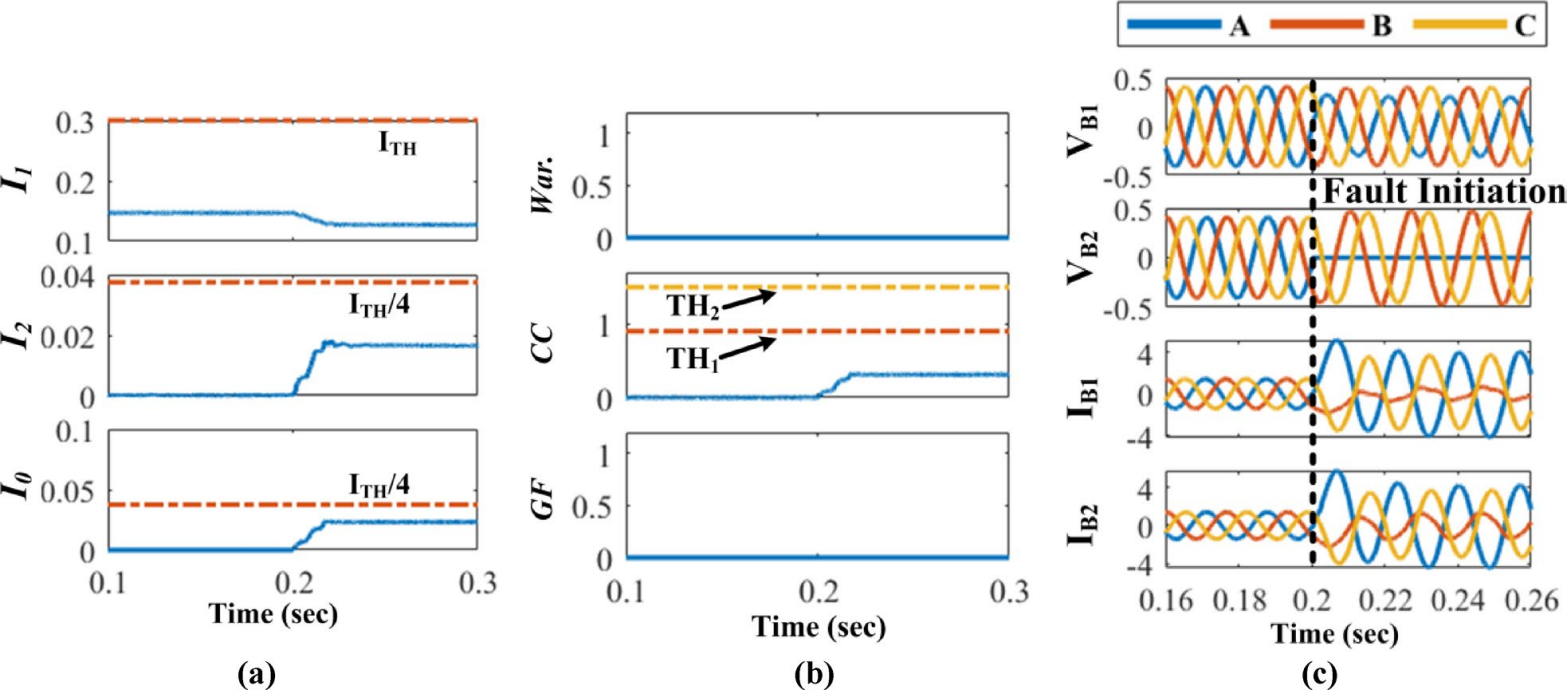
For an external bolted fault at Bus-2 (**a**) SSDC, (**b**) Relaying criterion coefficients, and (**c**) 3-phase voltages and currents at Bus-1 and Bus-2 respectively.

The most desirable property of a relaying system is its selectivity. To examine the performance of the proposed algorithm against this property; an external close-in bolted AG fault is introduced at B2, and the acquired result is shown in Fig. 13 below. It can be evaluated from Fig. 13c that during the fault the value of voltage at B2 becomes zero. Also, neither *I*_*0*_ nor *I*_*2*_ has crossed their respective threshold values as manifested in Fig. 13a, so *War.* is low (i.e. equal to 0). Hence the fault is identified as external fault.

### Analysis with real-time simulator

**Fig. 14:**
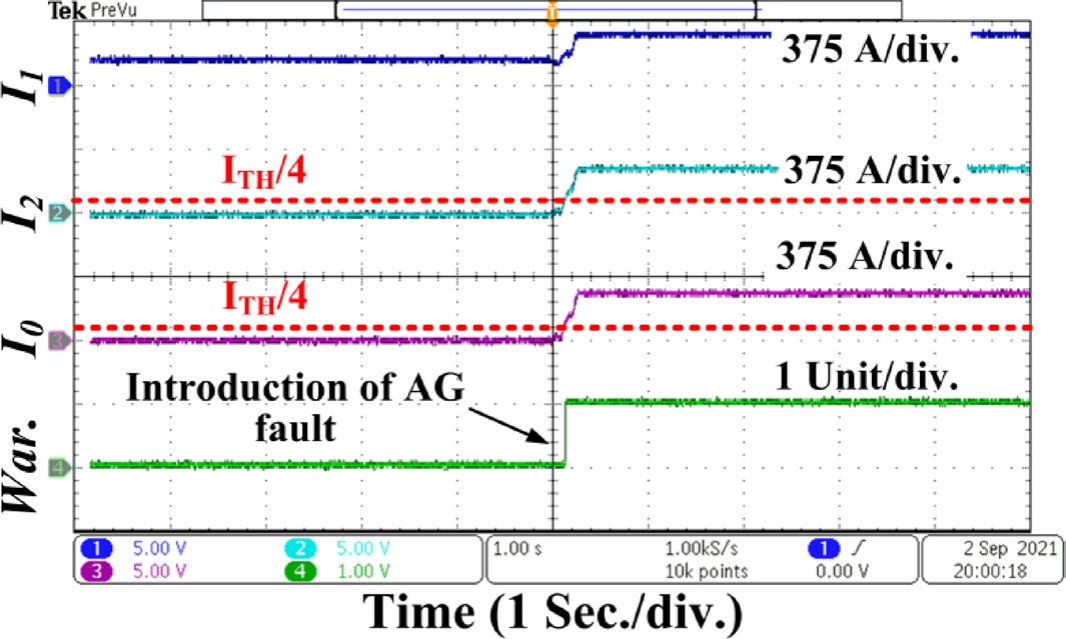
SDCCs and War. signal for an AG fault.

**Fig. 15:**
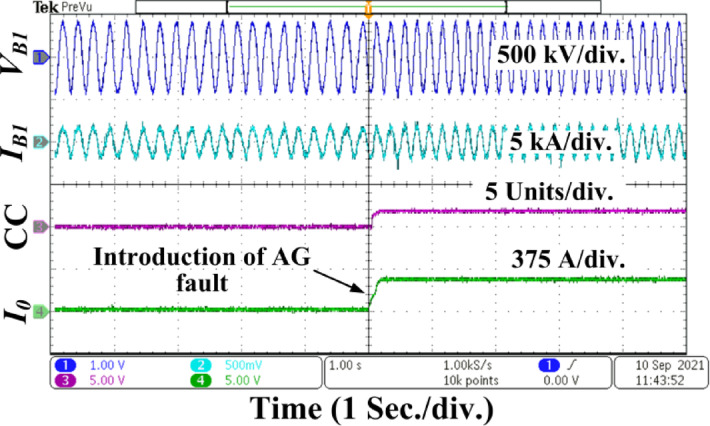
3-phase voltages and currents at Bus-1, relaying criterion coefficient, and *I*_*0*_ for an AG fault.

The performance of the scheme is also scrutinized and validated using Opal-RT 4510 real-time simulator for the practical application. An AG fault is introduced at 30 km away from B1 with *R*_*f*_ = 100 Ω and corresponding results are shown in Figs. 14 and 15 below. It can be seen from Fig. 14 that, all 3 SSDC are present; also *I*_*0*_ and *I*_*2*_ have crossed their respective threshold value, so *War.* is set as high (i.e. *War.* = 1). In Fig. 15, it can been seen that the magnitudes of phase-a voltage and current at B1 remain the same as before the fault, due to the high value of *R*_*f*_. However, the value of CC = 2 (As shown in Fig. 15, the CC scale is 5 units per division; therefore, after the fault, the CC value is 2), indicating an internal LG fault. So, it can be conclude that the scheme remains selective and accurate.

**Fig. 16:**
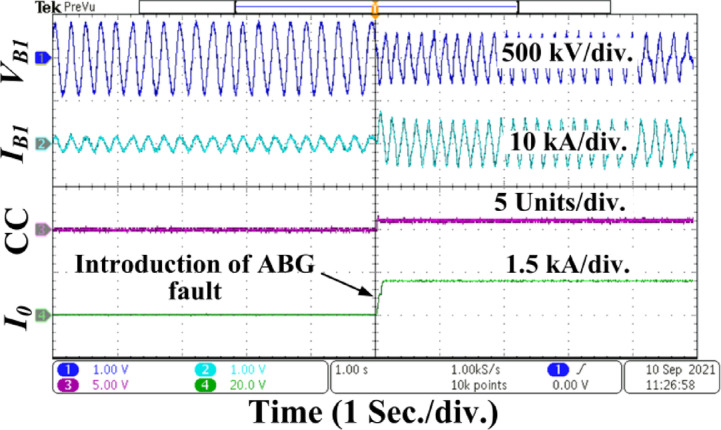
3-phase voltages and currents at Bus-1, relaying criterion coefficient, and *I*_*0*_ for an ABG fault.

An ABG fault is introduced at 30 km away from B1 with *R*_*f*_ = 10 Ω and corresponding result is shown in Fig. 16 below. It can be seen from Fig. 16 that, the value of CC is more than TH_1_ but less than TH_2_. So, the fault is recognized as 2-phase fault. To determine the ground’s involvement, the magnitude of *I*_*0*_ has been compared with the threshold (I_TH_/4), and found to be more than that. Hence, the fault is identified as an LLG fault.

**Fig. 17:**
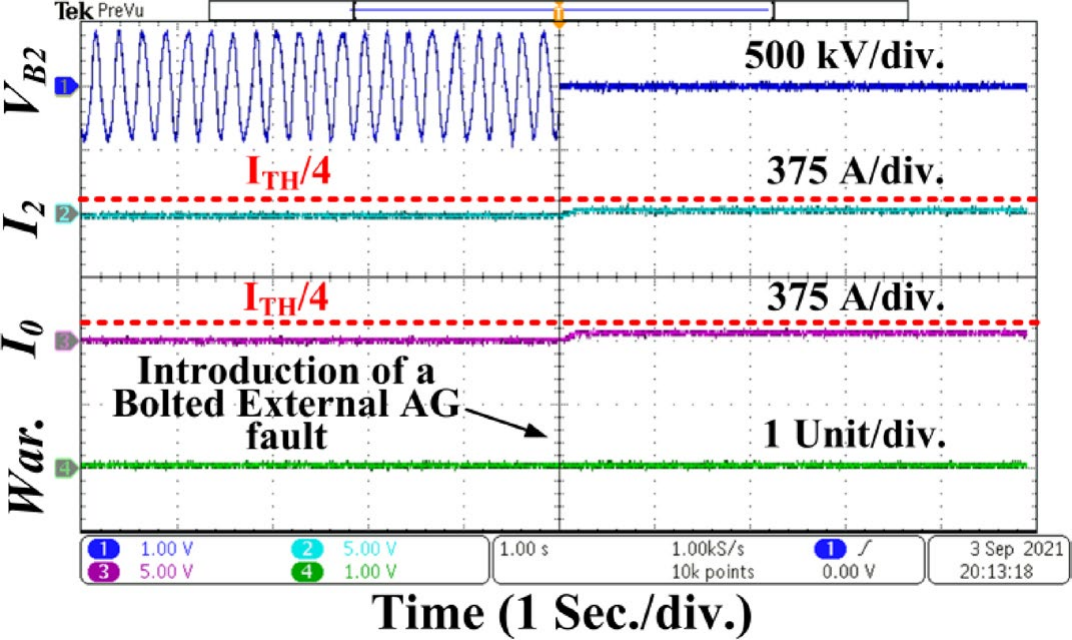
3-phase voltages at Bus-2, *I*_*2*_, *I*_*0*_, and *War.* signal for an external AG fault.

Similarly, for an external close-in 3-phase fault at B2, the obtained result is shown in Fig. 17. Due to the close-in low resistance 3-phase fault, the value of voltage at B2 becomes zero. Also, neither *I*_*0*_ nor *I*_*2*_ has crossed their respective threshold values, so *War.* is low (i.e. equal to 0). Hence the fault is identified as external fault.

### Comparative analysis

In order to showcase the silent features of the proposed scheme, some of the key features of the protection schemes available in the literature have been compared; and the comparative analysis are listed in the Table 6. It have been found that the algorithms were discussed in reference^19,28–30^ have not studied the impact of simultaneous faults, and faults with SSE. The impact of measurement noise were not discussed in references^28–30^. Moreover, real-time validations are missing in references^28,29^ and references^19,30^ require training for any change in the protection zone. Based on the analysis, the proposed algorithm found to be superior among the latest available UPFC protection schemes for the transmission lines in the literature.

**Table 6:** Comparative analysis incorporating the latest schemes available in the literature.

Relaying features	References				
	^19^	^28^	^29^	^30^	Proposed Algorithm
Max. Fault DT (ms)	ND	< 5	3	10	< 3.5
Max. FR considered (Ω)	200	100	100	100	100
Analysis with Simultaneous faults	✗	✗	✗	✗	✓
Performance with SSE	✗	✗	✗	✗	✓
Analysis with Measurement Noise	✓	✗	✗	✗	✓
Study with Dynamic compensation	✓	✓	✓	✓	✓
Requirement of Algorithm Training	✗	✓	✓	✗	✗
Real-Time Validations	✓	✗	✗	✓	✓

**ND = Not Discussed, ✗ = No, ✓ = Yes.

## Conclusion

A differential sequence current component-based protection scheme for an UPFC compensated TLN (end-of-line compensation) has been suggested in this article. The time-synchronized GPS voltage and current signals are used to calculate criterion coefficient (CC) for fault classification. A warning signal *War.* is used for internal fault detection. The proposed algorithm is evaluated and validated in the MATLAB Simulink environment for variations in FD, FR, and UPFC operating modes, Simultaneous faults, with SSE, and for different fault types as well. The scheme’s performance is also compared with latest available techniques in the literature; and diagnosed with the Opal-RT 4510 real-time simulator for the practical implementation. On evaluating the acquired results the scheme found to be accurate, selective, and robust for the protection of TLN with dynamic compensation.

## Data Availability

The datasets used and/or analyzed during the current study available from the first author on reasonable request.

## Appendix 1

*Generator data:*

G1-8500 MVA, Phase to phase voltage (Vrms) = 500e3*1.0491 kV, Frequency = 60 Hz, Phase (deg.) = 9.2.

G2-9000 MVA, Phase to phase voltage (Vrms) = 500e3*0.98 kV, Frequency = 60 Hz, Phase (deg.) = − 30.8.

G3-6500 MVA, Phase to phase voltage (Vrms) = 500e3*1.0 kV, Frequency = 60 Hz, Phase (deg.) = − 10.8.

*Transmission line data:*

**Table:**

Positive sequence resistance = 0.02546 Ω/km, Positive sequence inductance = 0.9337 mH/km, Positive sequence capacitive reactance = 12.74 nF/km,	Zero sequence resistance = 0.3864 Ω/km, Zero sequence inductance = 4.1264 mH/km, Zero sequence capacitance = 7.751 nF/km

*Load data*: 500 kV, 60 Hz, 200 MW.

*UPFC data*: A GTO-based UPFC is of 48-Pulse, 500 kV and 100 MVA rating.

**Table:**

*Shunt converter* MVA Rating = 100 MVA Primary Nominal Voltage = 500 kV Secondary Nominal Voltage = 15 kV	*Series converter* MVA Rating = 100 MVA Primary Nominal Voltage = 12.5 kV Secondary Nominal Voltage = 12.5 kV Percentage of voltage injection = 10%
